# Nonviral Gold Nanoparticle-Mediated Delivery of CRISPR-Cas9 Ribonucleoprotein and Long DNA Transgenes Into Primary Blood Cells

**DOI:** 10.1002/anbr.202500242

**Published:** 2026-03-31

**Authors:** Rachel A. Cunningham, Karthikeya S. V. Gottimukkala, Daniel D. Lane, Katrina Poljakov, Patricia Lipson, Mark R. Enstrom, Alessandro Rizzi, Aude G. Chapuis, Jennifer E. Adair

**Affiliations:** 1 Department of Laboratory Medicine and Pathology, University of Washington, Seattle, Washington, USA; 2 Translational Science and Therapeutics Division, Fred Hutchinson Cancer Center, Seattle, Washington, USA; 3 Department of Genetic and Cellular Medicine, University of Massachusetts Chan Medical School, Worcester, Massachusetts, USA

**Keywords:** CRISPR, gene therapy, immune engineering, nonviral delivery

## Abstract

Gene editing using CRISPR systems has gained traction for its potential to treat various diseases, but delivery remains a major challenge. We have previously reported an entirely synthetic gold nanoparticle formulation which can safely and effectively deliver multiple different CRISPR systems as ribonucleoprotein (RNP) into hematopoietic stem cells and immune cells. Here, we describe a modified version of this nanoparticle to simultaneously deliver CRISPR Cas9 RNP and transgene-encoding DNA templates (HDT) as long as ~2.1 kb. We evaluate this HDT-CRISPR-AuNP for gene editing at two loci of interest with different transgene cargo. These nanoparticles successfully deliver gene editing into primary human T cells and hematopoietic stem and progenitor cells with insertion of an antigen-specific T cell receptor transgene. This proof-of-concept immune engineering study demonstrates a simple, synthetic nanoformulation for co-delivery of all cargo required for CRISPR-mediated delivery of transgene cassettes with potential for efficacy in vivo.

## 1 | Introduction

CRISPR (clustered regularly interspaced short palindromic repeats) is a family of bacterial endonucleases that can be programmed to cut a particular site in double-stranded DNA. After conjugation to a cognate guide RNA (gRNA), CRISPR nucleases form functional ribonucleoprotein (RNP) complexes capable of mediating cutting with high specificity [1–3]. Since its discovery in 2012 [1], CRISPR has generated significant interest for its potential to engineer the human genome for health.

CRISPR-based genetic therapies have already revolutionized treatment of some common genetic disorders. The first authorized CRISPR therapy (exagamglogene autotemcel) utilizes ex vivo manipulation of patient hematopoietic stem and progenitor cells (HSPCs) to functionally cure sickle cell disease (SCD) [4], a serious condition which affects more than 8 million people worldwide [5]. An ongoing late-stage clinical trial is evaluating in vivo delivery of CRISPR mRNA lipid nanoparticles (LNPs) to liver hepatocytes for the treatment of transthyretin amyloidosis. This trial has reported promising preliminary results [6], further increasing enthusiasm for CRISPR gene therapies. While these successes bolster excitement for CRISPR genome engineering to transform human health, there are still significant barriers to widespread adoption. One of the most pressing problems to be solved is the delivery of multiple different types of gene editing cargo, particularly for in vivo delivery.

For certain use cases, multicomponent gene editing involving both CRISPR protein and DNA transgene templates may be necessary. Both examples of early CRISPR success above leverage the RNP complex alone to render site-specific mutations that provide therapeutic value for the target disease. Neither SCD nor transthyretin amyloidosis requires integration of new genes into the genome, and so delivery of RNP alone is sufficient for these patients. However, many potential disease indications require the insertion of novel transgenes to restore function or alleviate disease state, such as immune engineering of B cells for treating HIV [7] or T cell engineering for treating cancer [8]. Achieving CRISPR-mediated integration of new DNA large enough to encode transgenes into the human genome has proven difficult in an in vivo setting. In vivo delivery of gene therapy could be beneficial to access in low-resource settings where cost and complexity of ex vivo manufacturing and conditioning required prior to reinfusion represent significant barriers to patients. While ex vivo electroporation-based methods of transgene delivery offer relatively low cost, to date, the most widespread approach for transgene delivery with or without CRISPR has been dependent on viral vectors. These vectors—adeno-associated viruses (AAV) or lentivirus—must be assembled by living cells and then purified from cellular components, making cell therapy manufacturing complex and expensive [9]. AAV has demonstrated efficacy as an in vivo delivery platform for CRISPR but is limited by its packaging capacity of about 5 DNA kilobases (kb), making it difficult to ensure simultaneous delivery of CRISPR and transgene [9, 10]. Additionally, the inherent immunogenicity of viral vectors causes concern for potential redosing [9].

As a result of concerns about viral delivery, there is increasing interest in alternative synthetic delivery vehicles for delivery of gene therapies. Synthetic therapies offer potential inexpensive in vivo gene therapy [10], an attractive prospect for patients in low- and middle-come countries. Of the many synthetic nanoparticles under development, the most studied are LNPs following clinical success in the SARS-CoV2 pandemic as mRNA vaccines [11, 12]. Unsurprisingly, there is a great deal of interest in engineering LNPs for CRISPR delivery [10, 13]. LNPs are constructed of cationic lipids which favorably package negatively charged cargo such as oligonucleotides. As such, the most widespread use of LNPs for CRISPR delivery has been to package CRISPR cargo as nuclease mRNA and gRNA [10], avoiding combining complex cargo such as RNP and long DNA transgenes.

Here, we focus on delivery of CRISPR cargo to hematopoietic cells. Gene editing of HSPCs is relevant in many disease types, from infectious to malignant and carries unique challenges. Durable therapeutic value requires permanent integration of genetic changes into the genome such that these alterations are inherited during cell division, a hallmark of hematopoiesis. Previous work demonstrated delivery of CRISPR systems as RNP alongside DNA templates is both safe and effective in hematopoietic cells such as T cells [14] and HSPC [15].

Previously we described a first-generation CRISPR-AuNP which carried Cas9 or Cas12a RNP alongside a small (~80 nt) single-stranded DNA template. This nanoparticle co-opted the homology-directed DNA repair (HDR) pathway to insert the novel sequence encoded by the HDR DNA template (HDT) into the genome [16]. In this initial proof of concept formulation only gRNA cargo was covalently tethered to the AuNP core. All other cargo (nuclease protein, cationic polymer, and HDT) were loaded based on protein–RNA interactions (for RNP) or electrostatic interactions (for cationic polymer to support endosomal escape and HDT). While this formulation demonstrated some gene editing activity in primary human HSPC in vitro, results varied per nuclease used and were very poor for Cas9.

We later observed sloughing of electrostatically bound cargo upon exposure to culture conditions using confocal visualization with fluorescently tagged gRNA and HDT [17]. In addition, we characterized the suboptimal physiochemical effects on Cas9 with this assembly to develop an alternative formulation. Poor loading and activity of Cas9 could be overcome by preforming the RNP complex with cationic polymer and replacing 2K, branched polyethylenimine (PEI) with terminally thiolated, 10% polyethyleneglycol (PEG) grafted, low-molecular-weight (2K) branched chain polyethylenimine (PEI) (P10PSH) [17]. However, this second-generation CRISPR-AuNP carried insufficient cationic polymer to facilitate endosomal escape in primary human HSPC. To modify this particle design for increased endosomal escape, polyplexes were preformed with 20% PEG-grafted PEI (P20PSH) and assembled with concentrated AuNP cores. This third-generation CRISPR-AuNP can load different CRISPR nucleases, including Cas9, Cas12a, and a novel, engineered nuclease as RNP and demonstrated significant gene editing in primary human HSPC [18]. This fully synthetic, novel nanoparticle also showed initial safety and efficacy as a gene therapy when delivered systemically by two tail vein injections [18], suggesting potential for clinical translation. An inexpensive, redosable in vivo gene therapy would overcome the immunogenicity and delivery issues posed by AAV and LNPs, particularly if it could be shown to deliver multiple cargo types.

Here, we demonstrate functional third-generation nanoparticle capable of simultaneously packaging Cas9 RNP and long (2.1 kb) double-stranded DNA HDT (HDT-CRISPR-AuNP). By conjugating large, transgene-encoding HDT, we increase potential to mediate different types of therapeutic edits in vitro and potentially in vivo. We validate HDT-CRISPR-AuNP in primary human hematopoietic cells including T cells and HSPC. This nanoformulation can be synthesized in less than half a day with commercially available resources at low cost and is effective in primary human cells by simple addition to the culture medium, without the need for physical manipulation such as electroporation. Altogether, HDT-CRISPR-AuNP is a novel platform to simplify research and possibly clinical translation of novel therapeutic genome engineering strategies.

## 2 | Results

### 2.1 | Assembly of Monostable HDT-CRISPR-AuNP through Covalent Tethering to a Multilayer Third-Generation CRISPR-AuNP

**FIGURE 1 |:**
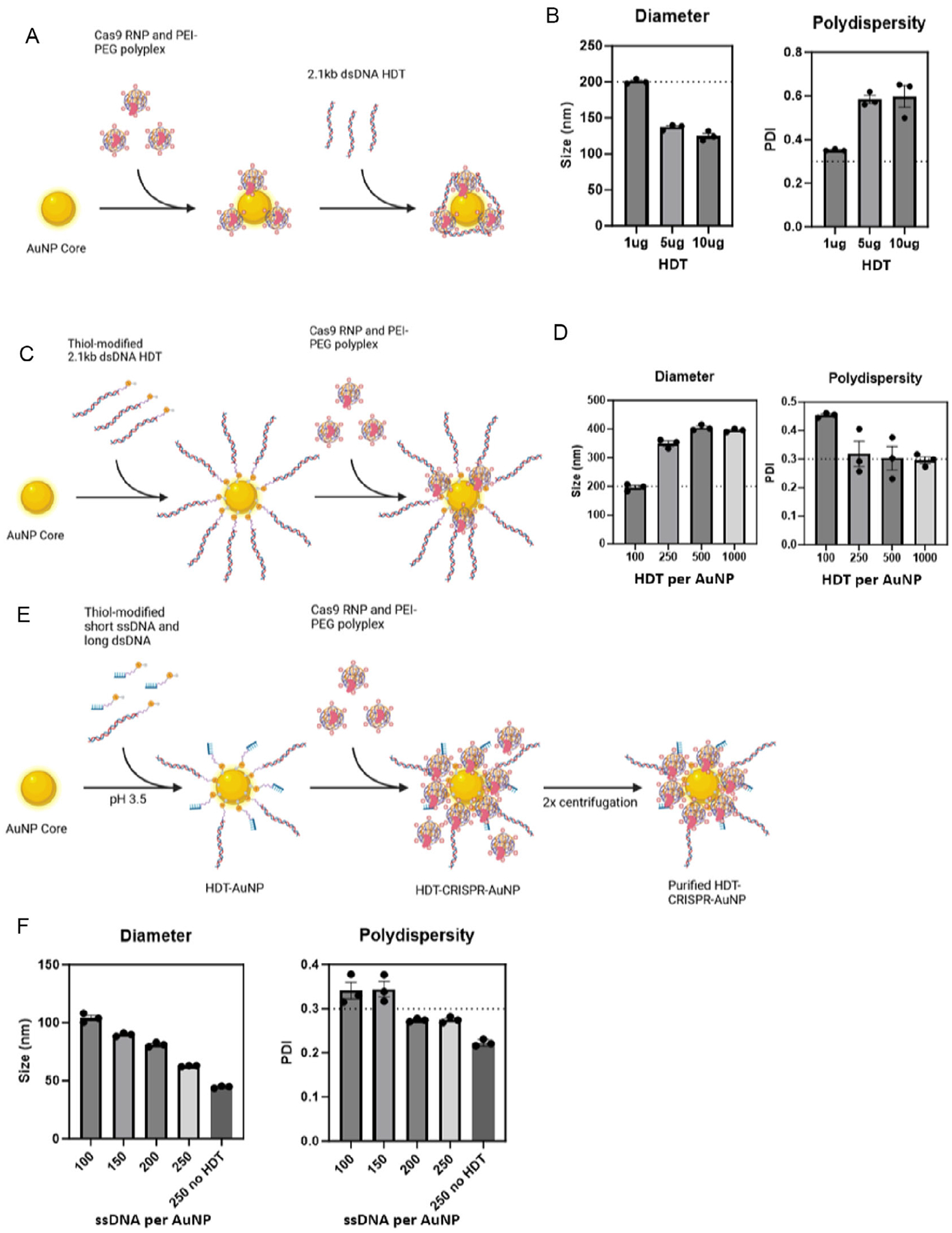
(A) HDT-CRISPR-AuNP synthesis was first attempted by formulating CRISPR-AuNP and decorating the nanoparticle surface with electrostatically bound HDT. Increasing the mass of dsDNA HDT from 1 to 10 μg per 20 μg AuNP resulted in (B) acceptable size but unacceptably high PDIs of above 0.3. (C) First forming a layer of thiol-modified HDT around the AuNP surface required calculating moles of HDT per moles of gold core. Ratios of 100 to 500 HDT molecules per core were tested, but all resulted in (D) unacceptable nanoparticle diameters of over 200 nm. (E) Surface stabilization of AuNP by the addition of a short, thiol-modified ssDNA in addition to thiol-modified HDT resulted in particles of an (F) acceptable diameter of under 100 nm and PDI of under 0.3 with 200 molecules of ssDNA and four molecules of HDT per AuNP core. Data represent three technical replicates (dots) as means (gray bars) ± standard error of the mean (error bars). Horizontal dashed lines represent the upper acceptable limits for size and PDI.

We first attempted to add a gene-length HDT to our third-generation CRISPR-AuNP via electrostatics (Figure 1A). We compared several concentrations of HDT per CRISPR-AuNP and assessed nanoparticle characteristics by dynamic light scattering (DLS). While each of the dsDNA titrations chosen resulted in acceptably sized nanoparticles (i.e., <200 nm hydrodynamic diameter), none demonstrated clinically acceptable polydispersity index (PDI) values of less than 0.3 [19] (Figure 1B). High PDI values suggest that AuNP core surface charges remain unsatisfied, resulting in aggregation of particles to achieve stability.

We previously demonstrated that binding of gRNA could satisfy AuNP core surface charges [16] in our first-generation CRISPR-AUNP. We hypothesized that formation of an inner layer of HDT covering the AuNP surface would permit a polyplex layer to bind and form covalent linkages to the gold core, trapping any loose DNA in the polymer with charge left unsatisfied by RNP.

A 2.1-kb dsDNA HDT encoding an enhanced green fluorescent protein (GFP) transgene flanked by homology arms to the human β−2-microglobulin locus (B2M) was synthesized by polymerase chain reaction (PCR) using a forward primer that contained a 12-carbon oligoethylene glycol (OEG) spacer with a 5′ terminal thiol. This 5′-SH-OEG-HDT was used to coat the gold core at different ratios of HDT molecules per individual AuNP (Figure 1C). We found that while higher ratios of HDT to AuNP resulted in monostable particles, the hydrodynamic diameters of these particles exceeded 200 nm, which is expected to reduce cellular entry [20, 21] (Figure 1D).

Since the stabilizing gRNA in our first-generation CRISPR-AuNP was short (~23 nt), we tried stabilizing the AuNP surface with a combination of short SH-OEG-ssDNA (25 nt), in addition 5′-SH-OEG-HDT (Figure 1E). This mixed-size DNA monolayering resulted in monostable nanoparticles with four HDT molecules and at least 200 ssDNA molecules per AuNP core (Figure 1F). The ratio of 4HDT/200ssDNA was chosen for further studies in order to maximize HDT loading.

### 2.2 | Characterization and Validation of Active Cargo on HDT-CRISPR-AuNP

**FIGURE 2 |:**
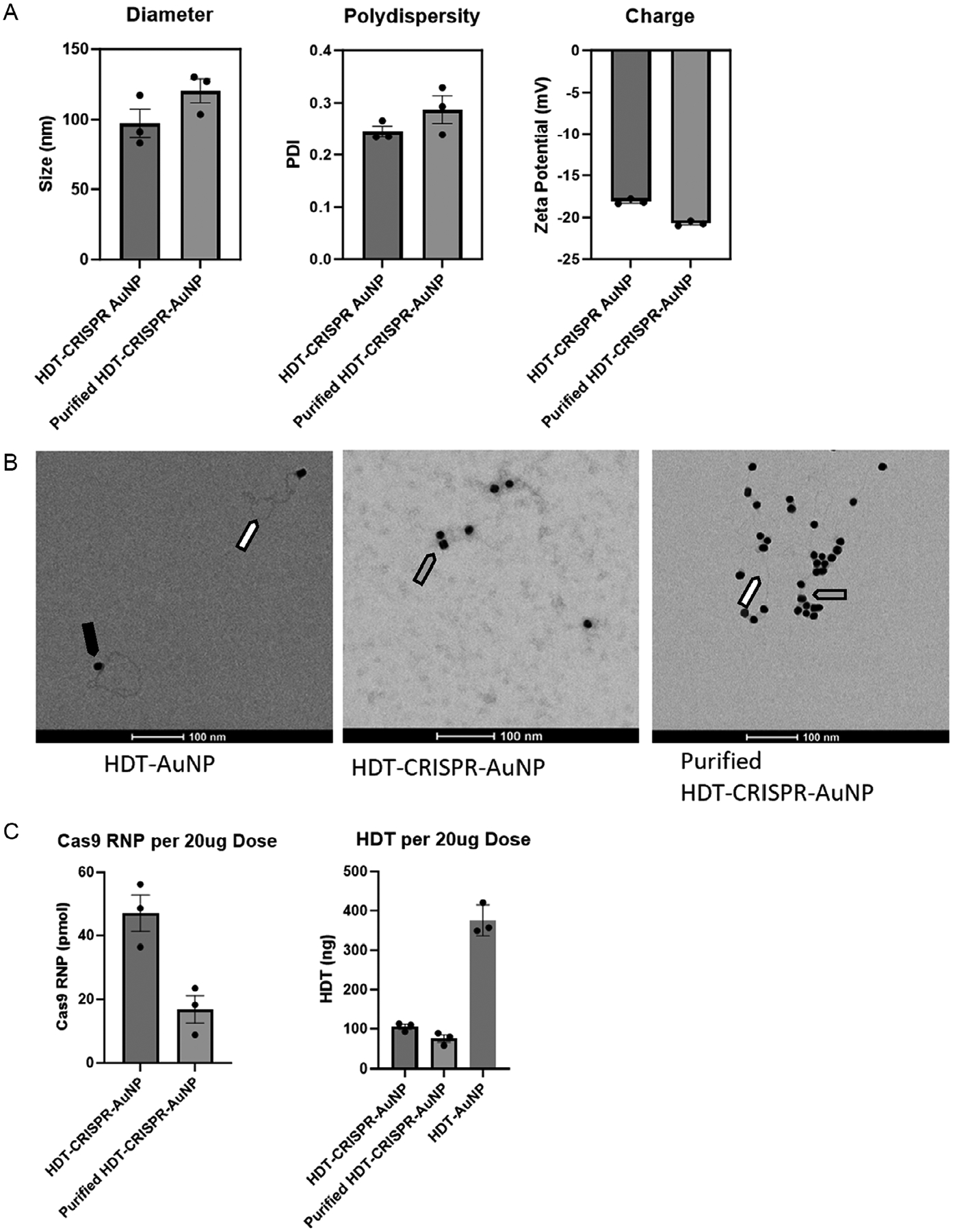
(A) DLS analysis of hydrodynamic diameter (left), PDI (middle), and zeta potential (i.e., surface charge) (right). Characterization of nanoparticles by DLS shows average size of 97.2 ± 18 nm for unpurified and 120.5 ± 14.72 nm for purified particles. Particles are acceptably monodisperse at PDIs of 0.245 ± 0.018 and 0.287 ± 0.046, respectively, and negatively charged at −18.1 ± 0.31 mV and −20.71 ± 0.29 mV. Data represent *n* = 3 separate nanoparticle syntheses per type. (B) TEM images of nanoparticles at 92000X magnification show attachment of long dsDNA (white arrows) to gold cores (black arrow), as well as polyplex coating (gray arrows) on fully formulated particles. (C) SDS–PAGE analysis to characterize nanoparticle active cargo loading shows on average 47.12 ± 9.93 pmol Cas9 RNP per 20 μg unpurified HDT-CRISPR-AuNP dose and 16.84 ± 7.48 pmol Cas9 RNP per 20 μg purified HDT-CRISPR-AuNP. Gel electrophoresis of nanoparticles treated with reducing agent BME to cause cargo release shows 105.1 ± 10.86 ng (81.84 ± 8.456 fmol) HDT per unpurified nanoparticle dose and 75.58 ± 15.46 ng (58.85 ± 12.04 fmol) per purified dose. The molar ratio of Cas9 RNP to HDT released from unpurified HDT-CRISPR-AuNP is 575.8 RNP per HDT, while purified HDT-CRISPR-AuNP release 286.15 RNP per HDT molecule. HDT-AuNP alone carried 375.25 ± 32.21 ng HDT indicating either significant displacement of the HDT from the gold surface by competing thiolated moieties, or sequestration of the HDT in the polymer matrix rendering it unavailable for charge-based gel imaging. Data represent three technical replicates (dots) as means (gray bars) ± standard error of the mean (error bars).

DLS analysis of nanoparticles formulated with 4 HDT and 200 ssDNA per core revealed monostability defined as mean hydrodynamic diameter of 97.2 ± 18 nm, PDI of 0.245 ± 0.018, and zeta potential of −18.1 ± 0.31 mV (Figure 2A).

We next evaluated stability after centrifugation to remove excipient cargo. The optimized nanoformulation maintained monostability after two rounds of spin purification, with a diameter increase to 120.5 ± 14.72 nm, PDI of 0.287 ± 0.046, and zeta potential of −20.71 ± 0.29 mV (Figure 2A). Transmission electron microscopy (TEM) images of HDT-CRISPR-AuNP stained with uranyl acetate show distinct attachment of long dsDNA to gold cores at the HDT-AuNP stage. Following complete assembly, we can also visualize polyplex corona and excess polymer in the unpurified stage, and a combination of long dsDNA and polyplex attachment to the gold core in the purified stage (Figure 2B).

For unpurified nanoparticles, Cas9 nuclease loading was determined by sodium dodecyl sulfate polyacrylamide gel electrophoresis (SDS–PAGE) to be 47.12 ± 9.93 pmol Cas9 and HDT loading was determined via agarose gel electrophoresis to be 105.1 ± 10.86 ng (81.84 ± 8.456 fmol) HDT per 20 μg AuNP dose (Figure 2C). Despite the increased hydrodynamic diameter observed following centrifugation, purified particles demonstrated 16.84 ± 7.48 pmol RNP with 75.58 ± 15.46 ng (58.85 ± 12.04 fmol) HDT. HDT-AuNP alone carried nearly 400 ng HDT indicating either significant displacement of the HDT from the gold surface by competing thiolated moieties or sequestration of the HDT in the polymer matrix rendering it unavailable for charge-based gel imaging (Figure 2C). The molar ratio of Cas9 RNP per HDT molecule decreased more than twofold after centrifuge purification, with unpurified HDT-CRISPR-AuNP releasing more than 575 RNP per each HDT, and purified nanoparticles releasing slightly over 286 RNP per HDT.

### 2.3 | HDT-CRISPR-AuNP Demonstrates Gene Editing in a T Cell Line

**FIGURE 3 |:**
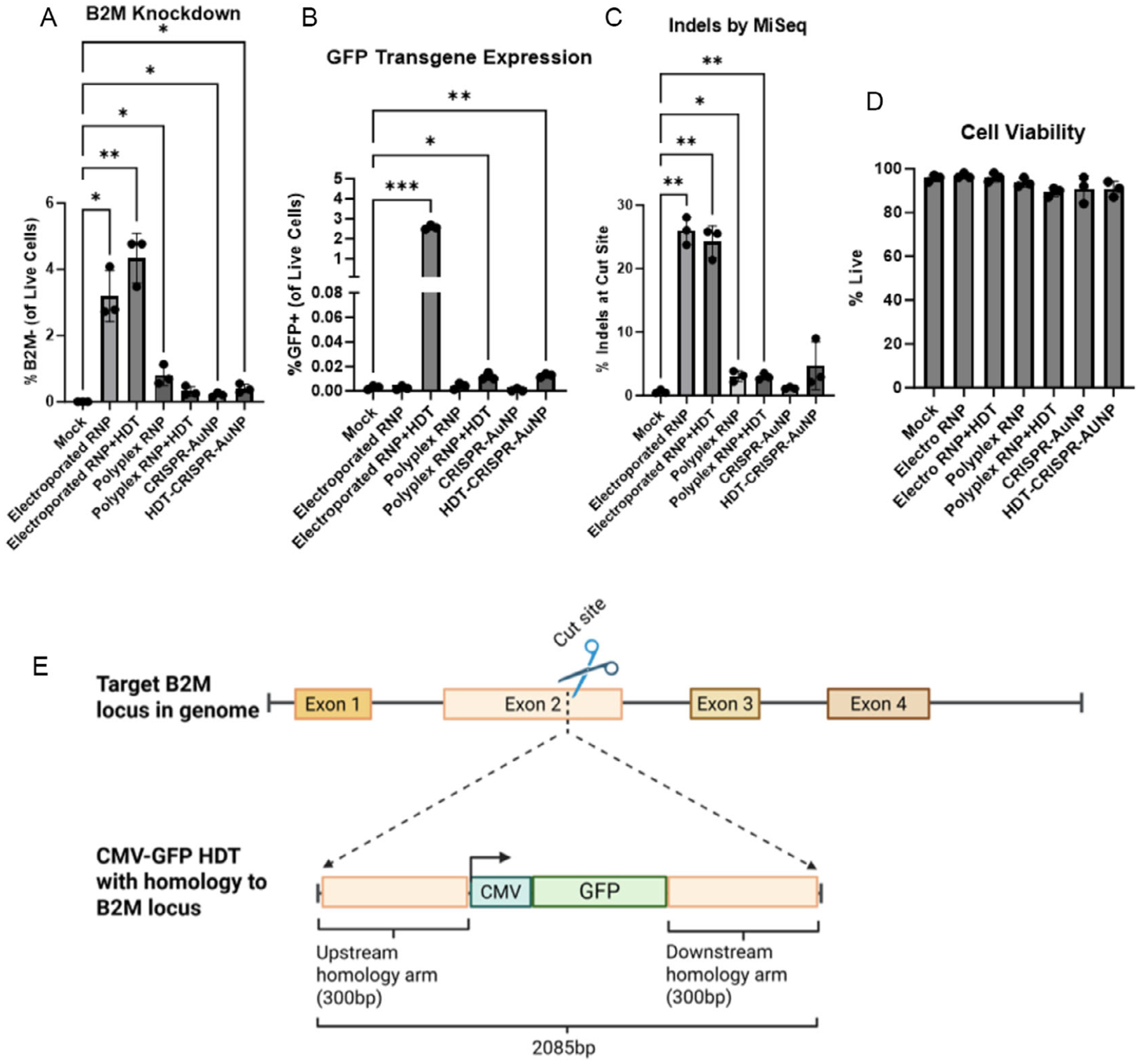
(A) Flow cytometry analysis of B2M knockdown in Jurkat cells. Cells treated with HDT-CRISPR-AuNP targeted to B2M and carrying a fluorescent reporter GFP transgene achieved significant B2M knockdown on par with non-HDT loaded CRISPR-AuNP across *n* = 3 technical replicates. Cells were harvested 72 h after nanoparticle treatment. (B) HDT-CRISPR-AuNP-mediated statistically significant expression of GFP reporter transgene as shown by flow cytometry. (C) Next-generation sequencing on gDNA isolated from Jurkat cells shows evident but not statistically significant indels at the B2M locus. (D) Cells remained highly viable after 72 h in culture with HDT-CRISPR-AuNP and other treatments. (E) Schematic of HDT design and insertion site in genomic B2M locus. Data represent three technical replicates (dots) as means (gray bars) ± standard error of the mean (error bars) with *p < 0.05, **p < 0.005, ***p < 0.0005.

To establish base efficacy of HDT-CRISPR-AuNP, unpurified HDT-CRISPR-AuNP carrying a GFP reporter transgene regulated by a minimal cytomegalovirus (CMV) promoter with homology arms to the B2M target locus cut site (Figure 3E) was tested in Jurkat cells. Jurkat cells are a human T cell line derived from acute T cell leukemia and are commonly used to evaluate CRISPR delivery prior to experiments in primary human T cells [22]. We and others have shown that CRISPR activity at the *B2M* locus, particularly in exon 2, results in mutations which in turn cause protein misfolding and loss of expression of B2M at the cell surface [18, 23]. A panel of B2M gRNAs were electroporated into cell lines and assessed for protein knockdown. The gRNA that resulted in the highest degree of B2M surface expression loss as measured by flow cytometry was carried forward for additional studies [18, 24]. We treated Jurkat cells with HDT-CRISPR-AuNP targeting *B2M* at a dose of 20 μg AuNP per million cells and analyzed resulting loss of B2M surface protein (B2Mlow/−) and gain of GFP protein expression (GFP+) by flow cytometry after 72 h in culture. Flow analysis showed significant B2M protein knockdown in cells treated with both CRISPR-AuNP and HDT-CRISPR-AuNP at 0.2% and 0.4% of live cells, respectively (Figure 3A), as well as statistically significant GFP transgene expression from both HDT-polyplex-treated and HDT-CRISPR-AuNP-treated cells at 0.012% and 0.013% of live cells, respectively (Figure 3B). Sequencing analysis revealed insertion and deletion (indel) mutations at the target *B2M* locus at 4.7% for cells treated with HDT-CRISPR-AuNP (Figure 3C), though not to a statistically significant extent. Importantly, nanoparticle treatment had no discernable effect on cell viability in this assay (Figure 3D).

### 2.4 | HDT-CRISPR-AuNP Efficacy in Primary Human Naïve T Cells

**FIGURE 4 |:**
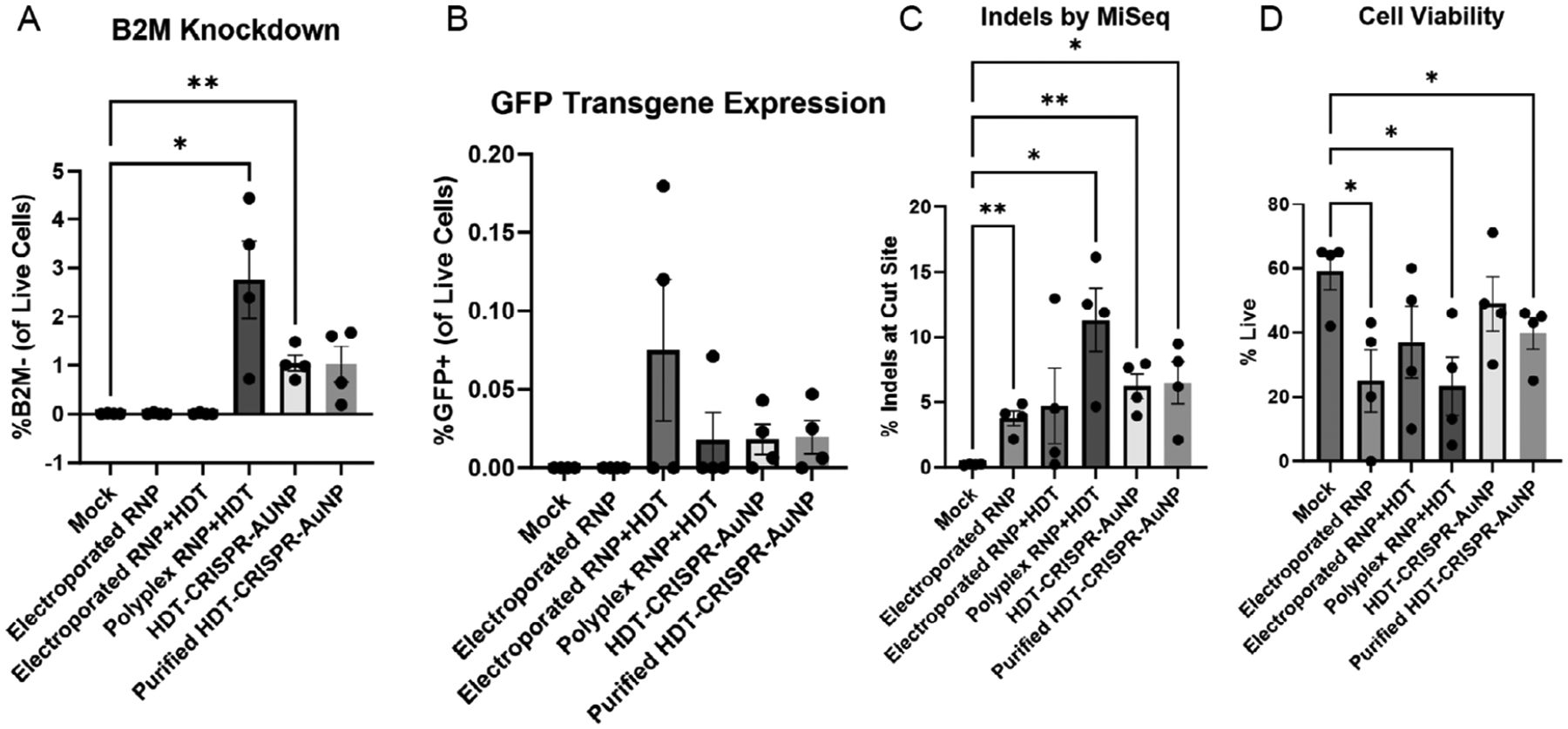
(A) Primary naïve T cells (*n* = 4) demonstrated slight but statistically significant B2M knockdown by flow cytometry when treated with unpurified but not purified HDT-CRISPR-AuNP. Cells were harvested 72 h after nanoparticle treatment. (B) T cells showed low but present expression of GFP reporter transgene from both non-purified and purified HDT-CRISPR-AuNP. (C) Sequencing analysis showed significant indels with upward of 5% editing at the B2M locus for both non-purified and purified nanoparticles. (D) Cell viability was low across all T cell samples and significantly lower than mock-treated cells for polyplex-treated and purified HDT-CRISPR-AuNP-treated samples. Data represent four biological replicates (dots) as means (gray bars) ± standard error of the mean (error bars) with *p < 0.05, **p < 0.005.

Following successful editing and transgene expression in Jurkat cells, the same *B2M* exon 2-targeted nanoparticle carrying a CMV-GFP reporter transgene was tested in primary human naïve T cells (CD3 + CD45RA + CD45RO-CD197+). Here, we tested both unpurified and purified HDT-CRISPR-AuNP to determine whether editing was a result of free polyplex in solution rather than assembled HDT-CRISPR-AuNP. Flow cytometry demonstrated evident, but not statistically significant B2M knockdown and GFP expression (Figure 4A,B), while sequencing results indicated statistically significant indel levels of 6.23 ± 1.90% and 6.49 ± 3.20% in cells treated with both unpurified and purified HDT-CRISPR-AuNP, respectively (Figure 4C). Cell viability was lower across all sample sets than observed in Jurkats (compare Figure 4D with Figure 3D), with purified, but not unpurified HDT-CRISPR-AuNP showing statistically significant differences in viability between nanoparticle-treated samples (49% for unpurified and 39.8% for purified) and mock-treated samples (59%) (Figure 4D).

### 2.5 | Efficacy in Primary Human T Cells at a Clinically Relevant Genomic Locus

We next sought to distinguish transgene expression in Jurkat and naïve T cells resulting from translation of exogenous double-stranded template DNA delivered into the cytoplasm from endogenously integrated HDT (the intended gene editing outcome). To this end, we formulated HDT-CRISPR-AuNP with a gRNA targeting the *T cell receptor alpha constant region* (*TRAC*) and an HDT encoding an antigen-specific TCR for the New York esophageal squamous cell carcinoma 1 (NY-ESO-1) neoantigen [14]. This gRNA and transgene combination was pioneered by Roth et al. and shown to be result in effective HDR-mediated template insertion when electroporated into primary T cells [14]. The transgene encoded in this HDT sequence consists of the full-length beta (*β*) chain and variable alpha (*α*) chain of the TCR, with no constitutive promoter included in the construct. Expression of the transgenic TCR will occur only when it is inserted correctly downstream of the endogenous TCR-*α* promoter [14]. HDT-CRISPR-AuNP synthesized with these novel elements were stable and monodisperse (Figure S2A).

**FIGURE 5 |:**
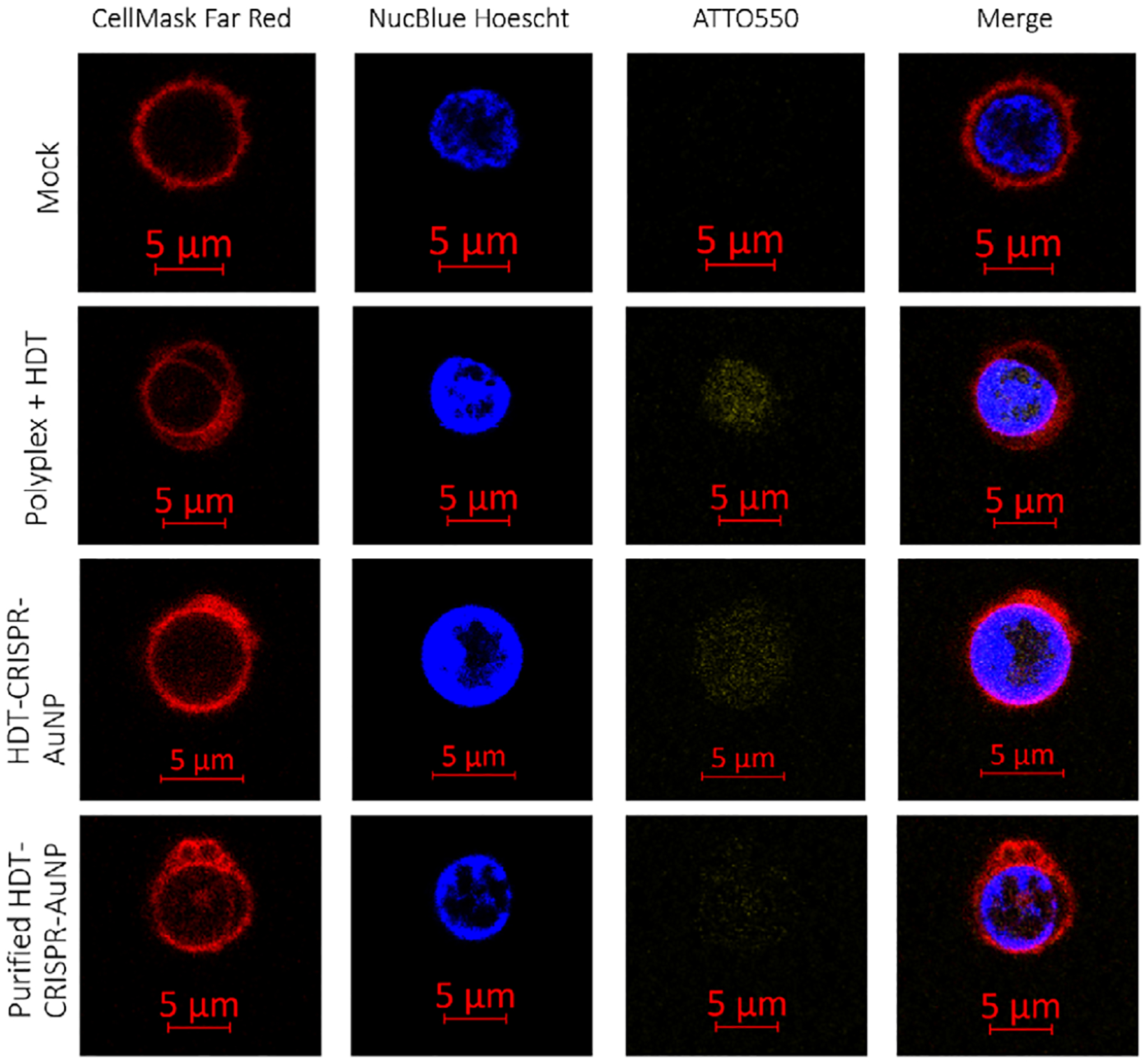
Confocal microscopy on T cells treated with fluorescently labeled HDT-CRISPR-AuNP shows diffuse cytosolic distribution and nuclear entry. Primary T cells treated with HDT-CRISPR-AuNP formulated using a tracrRNA tagged with ATTO550 (yellow) and labeled with CellMask Far Red Membrane Dye (red) and NucBlue Nuclear Stain (blue) were imaged at 64X by confocal microscope. Images representative of three biological replicates.

Having established that HDT-CRISPR-AuNP can be synthesized with modular HDT and gRNA cargo, we sought to determine where the nanoparticles trafficked in primary cells. Tracing cell uptake and cytosolic distribution lends us a better understanding of the mechanisms by which nanoparticles are endocytosed by the cells and therefore lets us know whether we are achieving cytosolic delivery or if any observed editing is the result of low-level leakage of gene editing cargo from intact endosomes. We synthesized HDT-CRISPR-AuNP with a fluorescent ATTO550 tag conjugated to the tracrRNA portion of the duplexed gRNA which colocalizes with nuclease in the RNP complex. Fluorescently tagged HDT-CRISPR-AuNP maintained stability as measured by DLS (Figure S2B). Confocal microscopy demonstrated diffuse ATTO550 fluorescence throughout the cell cytoplasm and nucleus, indicating that at least tracrRNA cargo are capable of cellular entry, broad cytosolic distribution, and nuclear entry (Figure 5), though treatment with purified nanoparticles results in lower fluorescence in the cells imaged in line with results from protein-loading characterization assays (Figure 2C).

**FIGURE 6 |:**
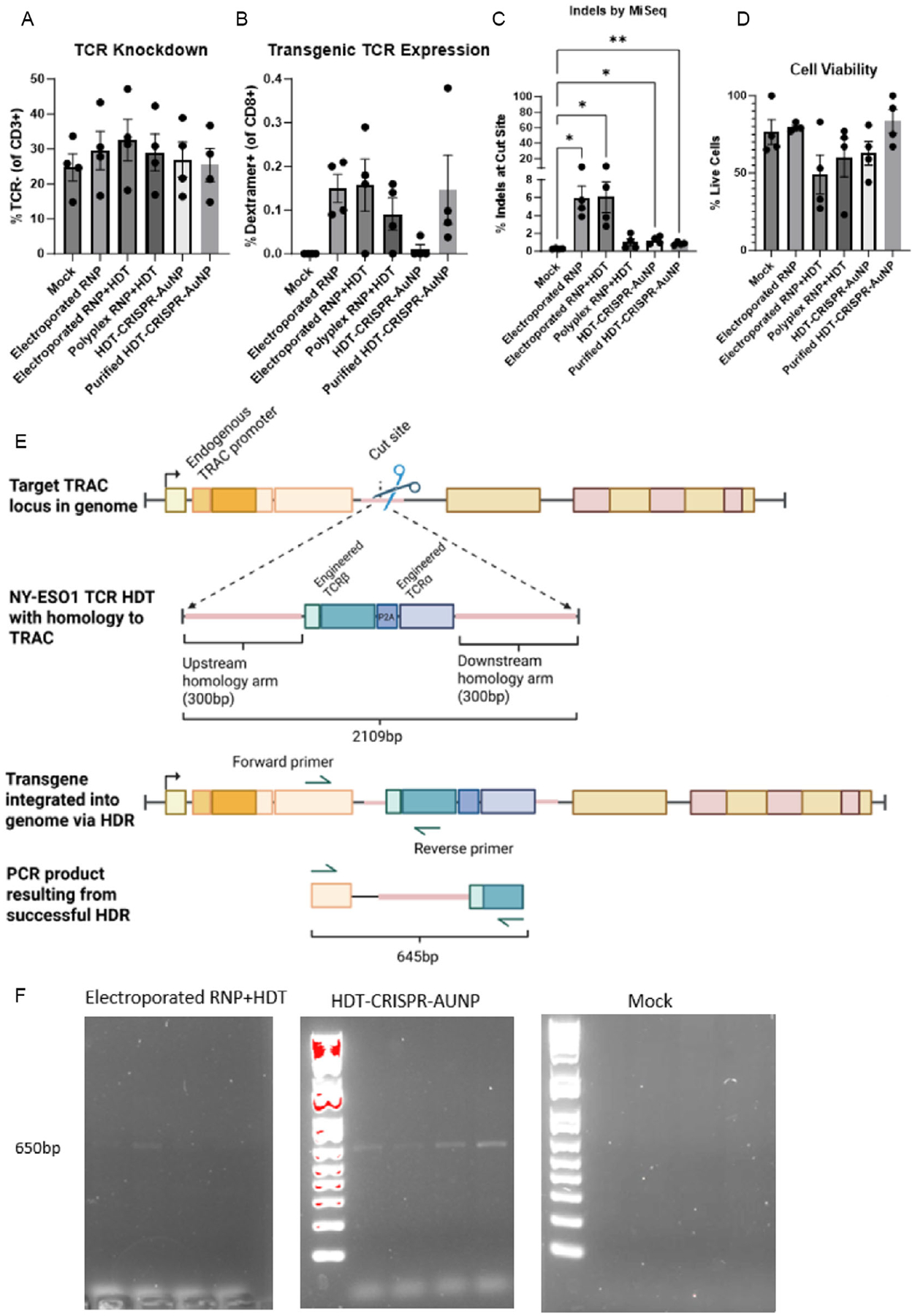
(A) Primary T cells (*n* = 4) treated with HDT-CRISPR-AuNP carrying Cas9 targeted to the TRAC locus and an HDT encoding a transgenic NY-ESO-1-specific TCR showed some TCR knockdown, but none was statistically significant. (B) CD8+ T cells showed no statistically significant binding of a PE-labeled type 1 MHC dextramer specific to the transgenic TCR. Results were confounded by high-background fluorescence as seen in electroporated RNP samples. (C) Sequencing analysis revealed statistically significant editing at the TRAC locus for both non-purified and purified nanoparticle samples with (D) no significant loss in viability. (E) NYESO-1 TCR construct contains a fully recombined TCRβ chain and linked TCRα chain. When inserted into the cut site by successful HDR, the expression of the engineered TCR is regulated by the endogenous TRAC promoter. PCR performed using a forward primer binding outside the homology arms of the HDT construct and a reverse primer within the variable β chain encoding sequence in the transgene resulted in (F) a PCR product of the expected size for positive control electroporated samples and for non-purified HDT-CRISPR-AuNP samples. No other sample set showed bands when run on an agarose gel. Data represent four biological replicates (dots) as means (gray bars) ± standard error of the mean (error bars) with *p < 0.05, **p < 0.005.

Non-fluorescently labeled HDT-CRISPR-AuNP was used to treat primary human T cells in culture. Following treatment with 20 μg AuNP equivalent of unpurified or purified HDT-CRISPR-AuNP, pan CD3+ T cells showed some knockout of endogenous TCR by both flow cytometry and next-generation sequencing (Figure 6A,C). However, dextramer binding to NY-ESO-1 TCR on the cell surface was not observed above background for any treated sample (Figure 6B). Notably, low dextramer binding was also observed in electroporated positive controls.

In the absence of confirmation of HDR by protein expression, we instead designed a PCR assay wherein the forward primer bound in the *TRAC* locus upstream of the 5′ homology arm of the HDT and the reverse primer bound within the variable beta chain sequence of the TCR transgene. PCR on a genomic DNA (gDNA) sample that contains the integrated insert should therefore yield 645bp product, while gDNA with no integrated HDT should yield no product (Figure 6E). Additionally, this assay cannot amplify exogenous HDT. PCR products from cells electroporated with RNP and HDT, as well as cells treated with unpurified HDT-CRISPR-AuNP, showed faint bands of the expected length (Figure 6F). Sanger sequencing of bands demonstrated upward of 95% homology to the target 645bp band (Figure S3), indicating successful, low level, integration of the transgene into the target locus. No other sample set, including RNP + HDT polyplex-treated cells and purified HDT-CRISPR-AuNP-treated cells, demonstrated visible PCR products in this assay (Figure S3).

### 2.6 | HDT-CRISPR-AuNP Efficacy in Primary Human CD34 + HSPC

**FIGURE 7 |:**
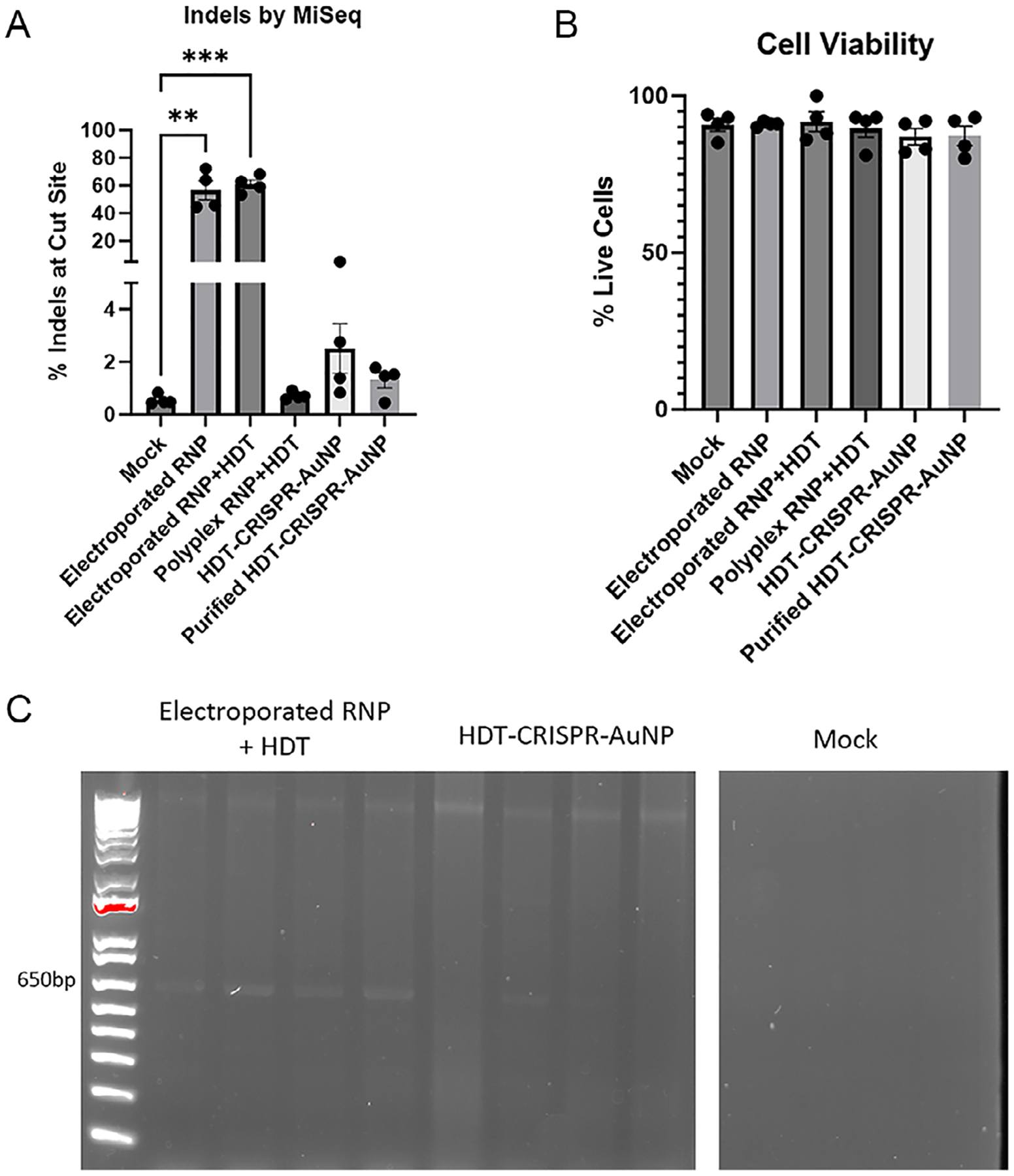
(A) CD34 + HSPC treated with TRAC-targeted HDT-CRISPR-AuNP showed high viability and (B) evident but not significant editing at the cut site for *n* = 4 nanoparticle-treated samples. (C) Integration PCR showed bands for electroporated control samples and two of four donors treated with non-purified HDT-CRISPR-AuNP. No other sample set showed bands. Data represent four biological replicates (dots) as means (gray bars) ± standard error of the mean (error bars) with *p < 0.05, **p < 0.005, and ***p < 0.0005.

*TRAC*-targeted HDT-CRISPR-AuNP carrying the NY-ESO-1 TCR construct were also used to treat primary human CD34 + HSPC in vitro. HSPCs do not express TCR, but several studies show that HSPCs edited with transgenic chimeric antigen receptors (CAR) express these transgenes after differentiation into a T cell lineage [25]. Endogenous TCRα promoter is inactive in HSPC, meaning no transgene expression would be expected on the surface of CD34+ cells even if HDR occurred. As such, gDNA was extracted from each sample and subjected to gDNA sequencing as well as the integration PCR assay we developed described above. Sequencing showed up to 5.08% indels at the *TRAC* locus for HDT-CRISPR-AuNP but fell short of statistical significance (Figure 7A).

Cell viability remained high regardless of treatment, with no statistical difference between mock-treated cells and any treated sample (Figure 7B). Integration PCR showed bands at the expected length for all four of the electroporated RNP + HDT samples and for two of the HDT-CRISPR-AuNP-treated samples (Figure 7C), with no bands for any of the other sample sets (Figure S3). Altogether these data indicate that HDT-CRISPR-AuNP is active in both primary human T cells and HSPC.

## 3 | Discussion

Developing an inexpensive technology that induces blood cells to express therapeutic transgenes is a goal that if reached has widespread implications for primary hematopoietic cell engineering at large. In addition to producing TCR-T and CAR-T cell products or creating a self-renewing reservoir of HSPCs that mature into transgenic T cells, such technology could be used to generate B cells that produce transgenic antibody, or plasma cells that generate any number of secreted therapeutic proteins [26–28]. A synthetic nonviral platform that can co-deliver Cas9 RNP and long transgenes into cells without the need for electroporation promises to overcome the dual barriers of cost and accessibility in CRISPR research and clinical development and translation. In contrast to other gold nanoparticle-based CRISPR delivery platforms, HDT-CRISPR-AuNP maintains monostability and acceptable size characteristics after formulation [29] and shows limited toxicity [29, 30]. HDT-CRISPR-AuNP does not require filtration after synthesis, maintains stability with simple centrifugation, and is capable of loading HDR templates of more than 2000bp, in contrast to other gold nanoparticle and LNP platforms [29, 31]. Synthesis is a cell-free process, unlike engineered virus-like particle (eVLP) and viral systems [32]. And finally, assembly of HDT-CRISPR-AuNP does not require protein engineering of the nuclease, in contrast to peptide-enabled RNP delivery for CRISPR engineering (PERC) and eVLP delivery systems [32, 33]. Relevant to PERC, HDT-CRISPR-AuNP can deliver more than a single RNP per particle, while both methods have demonstrated the benefit of covalently linking all cargo together for delivery in physiologic conditions [18, 33]. Importantly, HSPCs treated with HDT-CRISPR-AuNP maintain excellent viability and require less than 100pmol CRISPR RNP to mediate gene editing, as compared to the hundreds of pmol of CRISPR RNP reported for peptide-based delivery systems [33].

We have shown here that HDT-CRISPR-AuNP is capable of RNP and DNA co-delivery to purified cells in vitro, but the possibility of in vivo efficacy is a serious consideration. Currently, there are few FDA-approved gold nanomedicines, including PEG-functionalized gold nanorods used for tumor ablation [34]. Gold nanoparticles have been used in the past to treat inflammatory diseases like arthritis [35]. Short interfering RNA (siRNA)-coated AuNP has been shown to be well tolerated in a clinical trial for glioblastoma [36]. The potential of AuNP for in vivo efficacy as a gene editing platform has been shown by other research groups. Lee et al. demonstrated in vivo efficacy of RNP-functionalized gold nanoparticles in restoring function to a murine model of muscular dystrophy via HDR using a < 200bp DNA template [29]. The third-generation CRISPR-AuNP consisting of the gold core, CRISPR RNP, and thiolated PEI–PEG polymer without conjugated transgenes has shown little to no toxicity in vitro and an acceptable safety profile in immune-competent mice [18], indicating that HDT-CRISPR-AuNP may be capable of safely delivering DNA transgenes in addition to CRISPR gene editing in vivo.

A major shortcoming of HDT-CRISPR-AuNP shown here is low gene editing efficiency. dsDNA has been shown to cause some degree of cytotoxicity when delivered to the cell cytoplasm via electroporation [14], so simply increasing nanoparticle dose may result in higher toxicity along with higher transgene expression. It is likely that HDT-CRISPR-AuNP causes less toxicity than electroporation in most treated cell types due to the low concentration of dsDNA delivered, which in turn results in lower transgene expression than electroporated samples. While transgene expression from cells edited with HDT-CRISPR-AuNP is relatively low, in many contexts the transduction of only a few cells is sufficient to mediate an appropriate immune response. Studies in CAR-T cells show that a single clone dominates the expanded engineered cell population after infusion, indicating that a single successful transduction event is sufficient for clinical response in some contexts [37]. Research on stem cell engineering in murine models also suggests that successful HDR of a low number of CD34 + HSPC can result in production of an antibody transgene sufficient to mediate an immune response [15]. However, for disease indications beyond immune engineering where a small successfully edited B or T cell population can rapidly expand, HDT-CRISPR-AuNP requires further optimization. The low efficacy of transgene expression may be due to low dsDNA dose, inefficient delivery, or suboptimal chemistry. It is possible that use of PEGylated co-polymer shields dsDNA, reducing toxicity alongside efficacy [38]. Further optimization of nanoparticle cargo, including alternate CRISPR nucleases and transgene templates beyond dsDNA, may increase transgene integration efficiency.

The modularity of third-generation CRISPR-AuNP opens up the possibility to deliver engineered CRISPR systems capable of large transgene insertion without HDR, such as prime editors, or CRISPR-associated transposon (CAST) systems [39, 40]. So long as one of the nucleic acids involved is amenable to modification with a thiol-spacer group, engineered nucleases such as prime editors [39], as well as eeBxb1Prime-Assisted Site-Specific Integrase Gene Editing (eePASSIGE) and CAST -based gene editing systems [40, 41] should be compatible with CRISPR-AuNP synthesis. These would benefit from being delivered as RNP due to the complex and lengthy RNAs that comprise each of these systems [40, 41]. Conjugating highly optimized, engineered gene editing technologies to HDT-CRISPR-AuNP is likely to increase the efficacy of the platform for integrating large genes into target loci. Importantly, each of these systems is based on Cas9, which is limited to genomic regions containing the appropriate cognate protospacer-adjacent motif (PAM) sequence and unique spacer for limited off-target genomic activity. The ability to co-deliver HDT cargo will benefit diseases for which the intended genomic region does not contain these elements (upward of 95%) [42].

Continued research into gold-core nanoparticles and their ability to simultaneously deliver multiple cargo types into cells may yield valuable new treatments for inherited diseases, as well as non-inherited disorders which are candidates for gene therapy, while increasing the accessibility of gene therapy for all.

## 4 | Conclusion

In this proof of concept study, we demonstrated across two cell types and with two different transgenes the ability to passively deliver gene editing at two different loci by nonviral means and without electroporation using a synthetic nanoformulation which does not require protein engineering to assemble. While the efficiency of gene editing induced by HDT-CRISPR-AuNP is quite low compared to viral transduction or electroporation, existing data on the CRISPR-AuNP base platform suggest a high potential for in vivo use and at a fraction of the cost of viral therapies. Future studies in humanized mouse models may support the use of HDT-CRISPR-AuNP for in vivo stem cell and immune cell engineering.

## 5 | Methods

### 5.1 | Materials

All PCR primers and gRNAs were purchased from IDT (Coralville, Iowa, the United States). CMV-GFP reporter plasmids were synthesized by and purchased from GenScript USA (Piscataway, New Jersey, the United States). NY-ESO-1 TCR plasmid was a gift from Alexander Marson (Addgene plasmid # 207 482; http://n2t.net/addgene:207482; RRID:Addgene_20748). SpCas9 nuclease was purchased from Aldevron (Fargo, North Dakota, the United States). Chloroauric acid, sodium citrate dibasic trihydrate, hydrochloric acid, and β-mercaptoethanol (BME) were purchased from Sigma-Aldrich (St. Louis, Missouri, the United States). Neon Electroporation System 100 μL Kit (Invitrogen, Waltham, MA, the United States) was used on the Neon Transfection System (Invitrogen) for all electroporations. Flow cytometry antibodies were purchased from Biolegend (San Diego, California, the United States). MHC dextramer was purchased from Immudex (Copenhagen, Denmark). PEI–PEG copolymers were purchased from Nanosoft Polymers (Winston-Salem, NC, the United States). Other materials not listed were purchased through ThermoFisher Scientific (Waltham, Massachusetts, the United States).

### 5.2 | Nanoparticle Synthesis

Nanoparticle synthesis involves less than 2 h of hands-on time. AuNP of 15–20 nm in diameter is first synthesized using the Turkevich method [43] and then characterized via TEM (TALOS120C, ThermoFisher Scientific) and visible–ultraviolet (UV–Vis) absorbance at 520 nm wavelength (Nanodrop One, ThermoFisher Scientific) [16] to accurately estimate nanoparticle molarity for binding cargo. AuNP molarity was calculated using 1 cm Beer–Lambert law absorbance at 520 nm as previously described by Lane et, al [16]. AuNP is then incubated with thiol-oligoethylene glycol-modified HDT encoding a transgene construct with 300-bp homology arms (Table S1). HDT is added at a 4:1 molar ratio and a similarly modified 25 nt ssDNA at a 200:1 molar ratio in the presence of 25 mм citrate buffer. The short ssDNA coats the remainder of the gold surface and prevents aggregation of gold particles in the presence of citrate buffer. For the sake of replicability, different batches of AuNP were diluted to have equivalent molarities such that the amount of HDT loaded into a 20-μg dose of AuNP was consistently 4 μg dsDNA. After incubation with thiol-modified DNA, HDT-AuNP is washed twice by centrifugation at 15,000RCF at 4°C for 45 min at to remove unbound DNA. Meanwhile, 100 pmol Cas9 RNP is formed by combining Cas9 protein and thiol-modified gRNA at a 1:1 ratio. This thiolated RNP is combined with 10 μg 2000 W-graft-20 PEI–PEG thiolated polymer to form an RNP polyplex, by incubating at room temperature for 1 h prior to HDT-AuNP addition and vigorous trituration for 10 s. Final formulations were stored at 4°C for up to 3 days.

### 5.3 | Dynamic Light Scattering

Nanoparticle solutions were suspended in ultrapure water (UPW; Invitrogen) and analyzed in using a Zetasizer Nano ZS (Malvern Panalytical, Worcestershire, the United Kingdom). At least 5 μg gold solution was diluted into at least 100 μL UPW and loaded into 40 μL disposable cuvettes (BrandTech Scientific, Connecticut, the United States) for size and PDI measurement. Cuvettes were allowed to equilibrate for 1 min prior to reading with three averaged runs of 10 sub-runs each. For zeta potential measurement, nanoparticle solutions were diluted in 800 μL UPW and loaded into folded-capillary cuvettes (Malvern Panalytical). Charge measurements were taken using autosense. Measurements for each sample were taken in triplicate at 20°C.

### 5.4 | Imaging by Transmission Electron Microscopy

Fully formulated HDT-CRISPR-AuNP and HDT-AuNP were stained with 1% uranyl acetate as a negative stain to show protein, polymer, and DNA in high contrast. Grid-mounted nanoparticles were imaged using TALOS120C. For diameter calculations, TEM images were analyzed in ImageJ (Version: 1.54f) by measuring diameter on two axes for at least 20 AuNP.

### 5.5 | RNP Loading Characterization by Sodium Dodecyl Sulfate Polyacrylamide Gel Electrophoresis

A 2.5-μg CRISPR-AuNP solution based on gold core mass was combined with 4X lithium dodecyl sulfate (LDS) loading buffer (Invitrogen, Waltham, MA, the United States) according to the manufacturer’s instructions and incubated for 15 min at 95°C. Each sample was loaded into a NuPAGE 4%–12% Bis-Tris Mini Protein Gel (Invitrogen) and run at 200 mV for 30 min.

Gels were stained using SimplyBlue SafeStain (Invitrogen) imaged using iBright FL1500 Imaging System (Invitrogen) and analyzed for density using the iBright built-in gel analysis platform. Protein bands were selected and densities used to establish a standard curve for protein mass. Nanoparticle protein content was interpolated from the standard curve and calculated for a 20-μg CRISPR-AuNP dose.

### 5.6 | HDT Loading Characterization by Agarose Gel Electrophoresis

An 8-μg CRISPR-AuNP solution was incubated overnight in 5mм BME to cause release of surface-bound thiolated nanoparticle cargo. Released nanoparticle solution was combined with 10X BlueJuice gel loading buffer (ThermoFisher) and loaded into an agarose gel for electrophoresis. Samples were run for 45 min at 100 mV. Bands were analyzed using iBright and a standard curve established for band density. This was used to interpolate nanoparticle DNA content and calculated for a 20 μg dose.

### 5.7 | Jurkat Cell Culture

Jurkat cells (American Type Culture Collection, Manassas, VA, the United States) were thawed and plated in at 0.5 million cells per mL in Roswell Park Memorial Institute 1640 medium (RPMI, ThermoFisher) with 10% fetal bovine serum (FBS, ThermoFisher) and 1% penicillin streptomycin (ThermoFisher). After two passages in culture, cells were replated in serum-free, antibiotic-free RPMI, and serum-starved for 2 h. CRISPR-AuNP or polyplexes were added directly to cell media and incubated for 4 h before serum addition to media. Electroporated samples were electroporated using a Neon Transfection system (Invitrogen) according to manufacturer’s settings for Jurkats (1700 mV, 1 pulse, and 20 ms). Electroporated samples were treated with maximum possible RNP dose (100 pmol RNP) and HDT dose (400 ng HDT) and immediately placed into serum-containing RPMI. Cells were harvested for flow cytometry and gene editing analysis 72 h after nanoparticle treatment.

### 5.8 | Primary T Cell Culture

Primary peripheral blood naïve T cells and primary peripheral blood pan T cells were purchased from STEMCELL Technologies (Vancouver, Canada). Per STEMCELL, each donor’s written informed consent was obtained via IRB-approved consent forms and protocols. For each cell type, four unique donors were sourced, with half female and half male. Cells were thawed and placed into RPMI supplemented with 5 mм BME, 10% FBS (for naïve T cells) or 10% Human AB serum (Sigma-Aldrich) for pan T cells, and 1% penicillin streptomycin, 200 U mL^−1^ IL-2, 5 ng mL^−1^ IL-7, and 5 ng mL^−1^ IL-15 (ThermoFisher). Cells were harvested 72 h after nanoparticle treatment.

### 5.9 | CD34+ Cell Culture

Peripheral blood G-CSF-mobilized CD34+ blood stem cells were purchased from Core Center of Excellence in Hematology at the Fred Hutch following the protocol approved by the Fred Hutch Institutional Review Board (IRB) (protocol no. 985.03) and in accordance with the Declaration of Helsinki and the Belmont Report. Informed written consent was obtained from all human cell donors. Four unique cell donors were used: half female and half male. Cells were thawed and placed into Serum-Free Expansion Medium version II (SFEM II; STEMCELL Technologies) supplemented with 100 ng mL^−1^ human recombinant stem cell factor, Flt-3L, and thrombopoietin. Cells were rested overnight before being replated into non-supplemented Iscove’s Modified Dulbecco’s Medium (IMDM, ThermoFisher) for 2 h prior to treatment. Electroporated samples were electroporated according to the manufacturer’s instructions (1600 mV, 10 ms, and 1 pulse) and placed immediately into serum-supplemented stem cell media. Nanoparticle and polyplex samples were treated after 2 h of serum starvation and allowed to incubate for an additional 4 h. At 4 h, cells were supplemented with complete media and allowed to incubate a further 72 h before harvest for gene editing analysis.

### 5.10 | Cell Viability Measures

Cell viability was measured using a Countess Automated Cell Counter (Invitrogen). Cell samples were combined 1:1 with Trypan Blue (Invitrogen) prior to automated counting per manufacturer’s instructions.

### 5.11 | Confocal Microscopy

HDT-CRISPR-AuNP and HDT-polyplex were synthesized using Cas9 RNP formulated using ATTO550-labeled tracrRNA (IDT). Primary pan T cells were treated with nanoparticles or polyplexes as described previously. Cells were incubated with fluorescently labeled treatments for 4 h and then stained with CellMask Far Red membrane dye (ThermoFisher) and NucBlue Live Cell nuclear stain (Hoechst 33 342, ThermoFisher) according to manufacturer instructions. Cells were resuspended in 50 μL 1% FBS in 1X phosphate buffered saline (PBS, ThermoFisher) and imaged on a Zeiss LSM 780 confocal microscope (Carl Zeiss Microscopy, Oberkochen, Germany). Images were analyzed with Zen Blue (version: 3.1, ZEISS).

### 5.12 | Flow Cytometry

Antibody-fluorophore conjugates used in each flow cytometry experiment are detailed in Table S2. For each experiment, cells to be stained and analyzed were washed twice in 3 mL PBS to remove excess polymer and decrease the risk of antibody sequestration [44]. Washed cells were stained with Zombie Near Infrared Live/Dead stain (BioLegend) at a 1:500 dilution for 15 minutes. LiveDead stain was washed by centrifugation. For primary naïve and pan T cells, human FcR block (Miltenyi Biotec, Bergisch Gladbach, Germany) was incubated with cells according to manufacturer’ instructions for 15 min. Cells were washed and resuspended in FACS buffer (2% FBS in PBS) with the antibody cocktail detailed in Table SX for 45 min prior to final wash and acquisition. Cells were acquired on a BD FACSCelesta flow cytometer (BD Biosciences, Franklin Lakes, NJ, the United States).

### 5.13 | gDNA Extraction and Sequencing

Genomic DNA (gDNA) was extracted from cell samples using the Invitrogen Purelink Genomic DNA Isolation kit (ThermoFisher) according to manufacturer’s instructions. For MiSeq analysis of target cut sites, gDNA was amplified using MiSeq adapter primers designed in house for either the B2M or TRAC locus (Table S1). Amplicons were indexed and multiplexed using Nextera XT v2 indices (Illumina, San Diego, CA, the United States) and combined into sequencing libraries. Libraries were sequenced using paired end MiSeq. Resulting reads were analyzed for total gene editing and insertion/deletion mutations using an established in house analysis pipeline [16].

### 5.14 | Confirmation of Template Insertion by Polymerase Chain Reaction

PCR primers were designed such that the forward primer bound ~150bp upstream of the TRAC transgene HDT construct and the reverse primer within the variable beta chain of the transgenic TCR[cite] such that any product produced would have a size of 645bp. A 20-ng gDNA per sample was used in each PCR reaction with NEB Q5 HotStart 2x Mastermix (New England BioLabs, Ipswich, MA, the United States) according to manufacturer’s protocol. gDNA samples subjected to this PCR that yielded the expected band were subject to Sanger sequencing using the same forward primer to confirm band identity.

### 5.15 | Statistical Analysis

All statistical analyses were performed on raw data as Brown–Forsythe and Welch ANOVA tests. Analyses of nanoparticle characteristics (Figures 1, 2, S1, and S2) used *n* = 3 independently synthesized samples. Analysis of nanoparticle-treated cell lines (Figure 3) used *n* = 3 technical replicates per sample. Analysis of nanoparticle-treated primary T cells and HSPC (Figures 4–7) used *n* = 4 biological replicates from different donors. Graphpad Prism (10.0.4; Boston, MA, the United States) was used to present all results as the mean ± SEM. Statistical significance was defined as *p* ≤ 0.05.

## Supplementary Material

Supplemental Information

Additional supporting information can be found online in the Supporting Information section. **Supporting Figure S1**: Nanoparticle cargo loading gel images and standard curves. **Supporting Figure S2**: TRAC nanoparticle DLS for non-fluorescent (A) and ATTO550-labeled (B) particles. **Supporting Figure S3**: Integration PCR negative gels and Sanger sequencing analysis of positive bands. **Supporting Table S1**: Oligonucleotides. **Supporting Table S2**: Flow cytometry antibodies.

## Data Availability

The data that support the findings of this study are available from the corresponding author upon reasonable request.
