# Supplementary material for: Nonviral Gold Nanoparticle-Mediated Delivery of CRISPR-Cas9 Ribonucleoprotein and Long DNA Transgenes Into Primary Blood Cells
Source: Adv Nanobiomed Res. Author manuscript; Available in PMC 2026 May 5. (PMC13138722; doi:10.1002/anbr.202500242)
Supplement: Supplemental Information [file NIHMS2165002-supplement-Supplemental_Information.pdf]

Supporting Information

Supporting Information is available from the Wiley Online Library or from the author.

Non-viral Gold Nanoparticle Mediated Delivery of CRISPR-Cas9 Ribonucleoprotein and Long DNA Transgenes into Primary Blood Cells

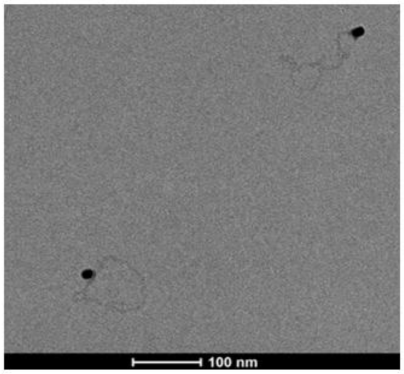

CRISPR/Cas9 has revolutionized the field of gene therapy, but delivery remains an outstanding issue. We propose a non-viral, gold-nanoparticle platform for co-delivery of CRISPR/Cas9 ribonucleoprotein and long, 2.1 kilobase dsDNA transgene constructs. These CRISPR-AuNP are inexpensive to produce and mediate gene editing and DNA delivery in T cells and CD34+ blood stem cells.

Supporting Information

Non-viral Gold Nanoparticle Mediated Delivery of CRISPR-Cas9 Ribonucleoprotein and Long DNA Transgenes into Primary Blood Cells

*Rachel A. Cunningham, Karthikeya S. V. Gottimukkala, Daniel D. Lane, Katrina Poljakov, Patricia Lipson, Mark R. Enstrom, Alessandro Rizzi, Aude G. Chapuis, Jennifer E. Adair**

Table S1: Oligonucleotides

Name	Oligonucleotide sequence
**HDT Sequences**	

B2M CMV-GFP HDT	GGGAAAGATACCAAGTCACGGTTTATTCTTCAAAATGGAGGTGGCTTGTTGGGAAGGTGGA<br>AGCTCATTTGGCCAGAGTGGAAATGGAATTGGGAGAAATCGATGACCAAATGTAAACACTTG<br>GTGCCTGATATAGCTTGACACCAAGTTAGCCCCAAGTGAAATACCCTGGCAATATTAATGTGTC<br>TTTTCCCGATATTCCTCAGGTACTCCAAAGATTCAGGTTTACTCACGTCATCCAGCAGAGAATG<br>GAAAGTCAAATTTCCTGAATTGCTATGTGTCTGGGTTTCATCCATCCGACACGTTACATAACTT<br>ACGGTAAATGGCCCGCCTGGCTGACCGCCCAACGACCCCCGCCCATTGACGTCAATAATGAC<br>GTATGTTCCCATAGTAACGCCAATAGGGACTTTCCATTGACGTCAATGGGTGGAGTATTTACG<br>GTAAACTGCCCACTTGGCAGTACATCAAGTGTATCATATGCCAAGTACGCCCCCTATTGACGTC<br>AATGACGGTAAATGGCCCGCCTGGCATTATGCCCAGTACATGACCTTATGGGACTTTCCTACTT<br>GGCAGTACATCTACGTATTAGTCATCGCTATTACCATGGTGATGCGGTTTTGGCAGTACATCAA<br>TGGGCGTGGATAGCGGTTTGACTCACGGGGATTTCCAAGTCTCCACCCCATTGACGTCAATG<br>GGAGTTTGTTTTGGCACCAAAATCAACGGGACTTTCCAAAATGTCGTAACAACTCCGCCCCAT<br>TGACGCAAATGGGCGGTAGGCGTGTACGGTGGGAGGTCTATATAAGCAGAGCTGGTTTAGT<br>GAACCGTCAGATCCGCTAGCGCTACCGGTCGCCACCATGGTGAGCAAGGGCGAGGAGCTGT<br>TCACCGGGGTGGTGCCCATCCTGGTCGAGCTGGACGGCGACGTAAACGGCCACAAGTTCAG<br>CGTGTCCGGCGAGGGCGAGGGCGATGCCACCTACGGCAAGCTGACCCTGAAGTTCATCTGC<br>ACCACCGGCAAGCTGCCCGTGCCCTGGCCCACCCTCGTGACCACCCTGACCTACGGCGTGCA<br>GTGCTTCAGCCGCTACCCCGACCACATGAAGCAGCACGACTTCTTCAAGTCCGCCATGCCCG<br>AAGGCTACGTCCAGGAGCGCACCATCTTCTTCAAGGACGACGGCAACTACAAGACCCGCGC<br>CGAGGTGAAGTTCGAGGGCGACACCCTGGTGAACCGCATCGAGCTGAAGGGCATCGACTTC<br>AAGGAGGACGGCAACATCCTGGGGCACAAGCTGGAGTACAACTACAACAGCCACAACGTCT<br>ATATCATGGCCGACAAGCAGAAGAACGGCATCAAGGTGAACTTCAAGATCCGCCACAACATC<br>GAGGACGGCAGCGTGCAGCTCGCCGACCACTACCAGCAGAACACCCCCATCGGCGACGGCC<br>CCGTGCTGCTGCCCGACAACCACTACCTGAGCACCCAGTCCGCCCTGAGCAAAGACCCCAAC<br>GAGAAGCGCGATCACATGGTCCTGCTGGAGTTCGTGACCGCCGCCGGGATCACTCTCGGCAT<br>GGACGAGCTGTACAAGTAGAGCGGCCGCGGGGATCCAGACATGATAAGATACATTGATGAG<br>TTTGGACAAACCACAACTAGAATGCAGTGAAAAAAATGCTTTATTTGTGAAATTTGTGATGCT<br>ATTGCTTTATTTGTAACCATTATAAGCTGCAATAAACAAGTTAACAACAACAATTGCATTCATTT<br>TATGTTTCAGGTTCAGGGGGAGGTGTGGGAGGTTTTTTAAGATTGAAGTTGACTTACTGAA<br>GAATGGAGAGAGAATTGAAAAAGTGGAGCATTCAGACTTGTCTTTCAGCAAGGACTGGTCT<br>TTCTATCTCTTGTACTACACTGAATTCACCCCCACTGAAAAAGATGAGTATGCCTGCCGTGTGA<br>ACCATGTGACTTTGTCACAGCCCAAGATAGTTAAGTGGGGTAAGTCTTACATTCTTTTGTAAG<br>CTGCTGAAAGTTGTGTATGAGTAGTCATATCATAAAGCTGCTTTGATATAAAAAAGGTCTATGG<br>CCATACTACCCTGAATGAGTCCCATCC
TRAC NY-ESO-1 TCR HDT	TTTCAGGTTTCCTTGAGTGGCAGGCCAGGCCTGGCCGTGAACGTTCACTGAAATCATGGCCT<br>CTTGGCCAAGATTGATAGCTTGTGCCTGTCCCTGAGTCCCAGTCCATCACGAGCAGCTGGTTT<br>CTAAGATGCTATTTCCCGTATAAAGCATGAGACCGTGACTTGCCAGCCCCACAGAGCCCCGCC<br>CTTGTCCATCACTGGCATCTGGACTCCAGCCTGGGTTGGGGCAAAGAGGGAAATGAGATCAT<br>GTCCTAACCCTGATCCTCTTGTCCCACAGATATCCAGAACCCTGACCCTGCCTCCGGATCCGGA<br>GAGGGCAGGGGATCTCTCCTTACTTGTGGCGACGTGGAGGAGAACCCCGGCCCCATGAGCA<br>TCGGCCTCCTGTGCTGTGCAGCCTTGTCTCTCCTGTGGGCAGGTCCAGTGAATGCTGGTGTCA<br>CTCAGACCCCAAAATTCCAGGTCCTGAAGACAGGACAGAGCATGACACTGCAGTGTGCCCA<br>GGATATGAACCATGAATACATGTCCTGGTATCGACAAGACCCAGGCATGGGGCTGAGGCTGA<br>TTCATTACTCAGTTGGTGCTGGTATCACTGACCAAGGAGAAGTCCCCAATGGCTACAATGTCT

	CCAGATCAACCACAGAGGATTTCCCGCTCAGGCTGCTGTCGGCTGCTCCCTCCCAGACATCTG<br>TGTACTTCTGTGCCAGCAGTTACGTCGGGAACACCGGGGAGCTGTTTTTTGGAGAAGGCTCT<br>AGGCTGACCGTACTGGAGGACCTGAAAAACGTGTTCCCACCCGAGGTCGCTGTGTTTGAGC<br>CATCAGAAGCAGAGATCTCCCACACCCAAAAGGCCACACTGGTATGCCTGGCCACAGGCTTC<br>TACCCCGACCACGTGGAGCTGAGCTGGTGGGTGAATGGGAAGGAGGTGCACAGTGGGGTC<br>AGCACAGACCCGCAGCCCCTCAAGGAGCAGCCCGCCCTCAATGACTCCAGATACTGCCTGAG<br>CAGCCGCCTGAGGGTCTCGGCCACCTTCTGGCAGAACCCCCGCAACCACTTCCGCTGTCAAG<br>TCCAGTTCTACGGGCTCTCGGAGAATGACGAGTGGACCCAGGATAGGGCCAAACCCGTCAC<br>CCAGATCGTCAGCGCCGAGGCCTGGGGTAGAGCAGACTGTGGCTTCACCTCCGAGTCTTACC<br>AGCAAGGGGTCCTGTCTGCCACCATCCTCTATGAGATCTTGCTAGGGAAGGCCACCTTGTATG<br>CCGTGCTGGTCAGTGCCCTCGTGCTGATGGCTATGGTCAAGAGAAAGGATTCCAGAGGCCG<br>GGCCAAGCGGTCCGGATCCGGAGCCACCAACTTCAGCCTGCTGAAGCAGGCCGGCGACGT<br>GGAGGAGAACCCCGGCCCCATGGAGACCCTCTTGGGCCTGCTTATCCTTTGGCTGCAGCTGC<br>AATGGGTGAGCAGCAAACAGGAGGTGACGCAGATTCCTGCAGCTCTGAGTGTCCCAGAAG<br>GAGAAAACTTGGTTCTCAACTGCAGTTTCACTGATAGCGCTATTTACAACCTCCAGTGGTTTA<br>GGCAGGACCCTGGGAAAGGTCTCACATCTCTGTTGCTTATTCAGTCAAGTCAGAGAGAGCAA<br>ACAAGTGGAAGACTTAATGCCTCGCTGGATAAATCATCAGGACGTAGTACTTTATACATTGCA<br>GCTTCTCAGCCTGGTGACTCAGCCACCTACCTCTGTGCTGTGAGGCCCCTGTACGGAGGAAG<br>CTACATACCTACATTTGGAAGAGGAACCAGCCTTATTGTTCATCCGTATATCCAGAACCCTGAC<br>CCTGCGGTGTACCAGCTGAGAGACTCTAAATCCAGTGACAAGTCTGTCTGCCTATTCACCGAT<br>TTTGATTCTCAAACAAATGTGTCACAAAGTAAGGATTCTGATGTGTATATCACAGACAAAACTG<br>TGCTAGACATGAGGTCTATGGACTTCAAGAGCAACAGTGCTGTGGCCTGGAGCAACAAATCT<br>GACTTTGCATGTGCAAACGCCTTCAACAACAGCATTATTCCAGAAGACACCTTCTTCCCCAGC<br>CCAGGTAAGGGCAGCTTTGGTGCCTTCGCAGGCTGTTTCCTTGCTTCAGGAATGGCCA
**PCR Primers**	
B2M HDT Forward Primer (FP)*	/5ThioMC6-D/iSp18/GGGAAAGATACCAAGTCACGGTTTA
B2M HDT Reverse Primer (RP)	GGATGGGACTCATTCAGGGTAGTAT
B2M MiSeq FP	GACACCAAGTTAGCCCCAA
B2M MiSeq RP	CATTCAGGGTAGTATGGCCATAG
TRAC HDT FP	/5ThioMC6-D/iSp18/TTTCAGGTTTCCTTGAGTGGC
TRAC HDT RP	TGGCCATTCCTGAAGCAAGGAAACAG
TRAC	CCATCACGAGCAGCTGGTTTCTAAGATGCTA

MiSeq FP	
TRAC MiSeq RP	CTGTTGCTCTTGAAGTCCATAGACCTCATGT
TRAC Integration PCR FP	TTCTGCTAATGCCCAGCCTAAG
TRAC Integration PCR RP	TGTCTTCAGGACCTGGAATTTTG
**gRNA Sequences**	
Cas9 B2M gRNA	/5ThioMC6-D/iSp18/rCrArGrUrArArGrUrCrArArCrUrUrCrArArUrGrUrGrUrUrUrUrArGrArGrCrUrArUrGrCrU
Cas9 TRAC gRNA	/5ThioMC6-D/iSp18/rArGrArGrUrCrUrCrUrCrArGrCrUrGrGrUrArCrAGrUrUrUrUrArGrArGrCrUrArUrGrCrU

*B2M HDT FP also functions as 25nt SH-OEG-ssDNA for HDT-CRISPR-AuNP synthesis

Table S2: Flow cytometry antibodies

Antibody	Clone	Fluorochrome	Dilution
B2M	2M2	PE	1:20
CD3	UCHT1	AF700	1:25
CD4	RPA-T4	V450	1:25
CD8	RPA-T8	APC	1:20
TCRab	IP26	PerCP-Cy5.5	1:20

Figure S1: Nanoparticle cargo loading gel images and standard curves

A. SDS-PAGE gel images and standard curves

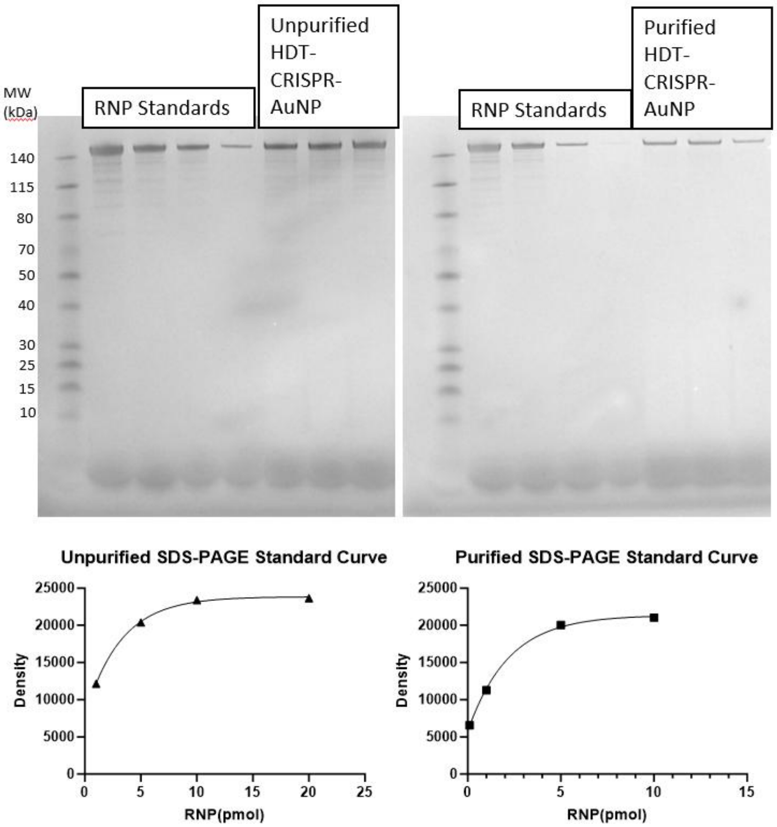

B. HDT loading gel images and standard curve

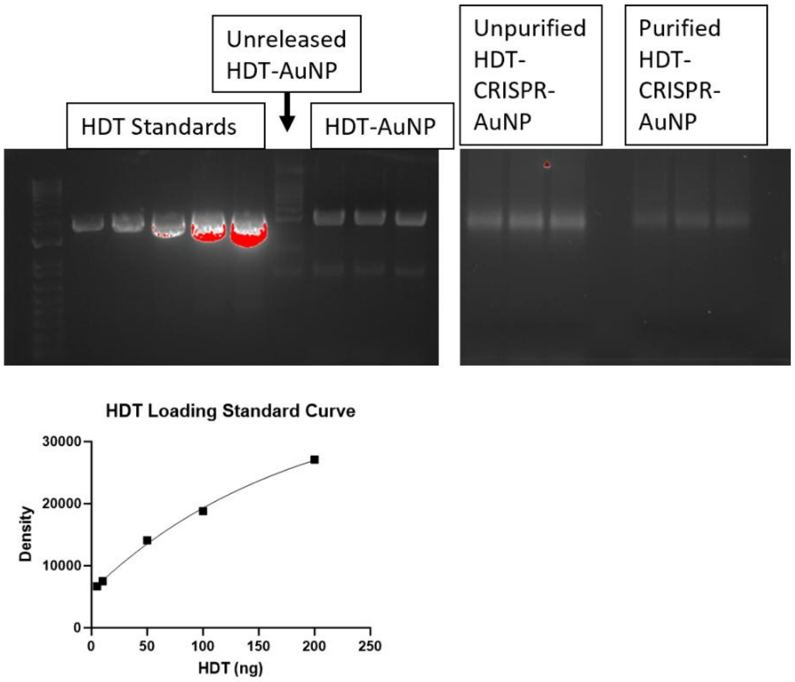

Figure S2: TRAC nanoparticle DLS for non-fluorescent (A) and ATTO550-labeled (B) particles

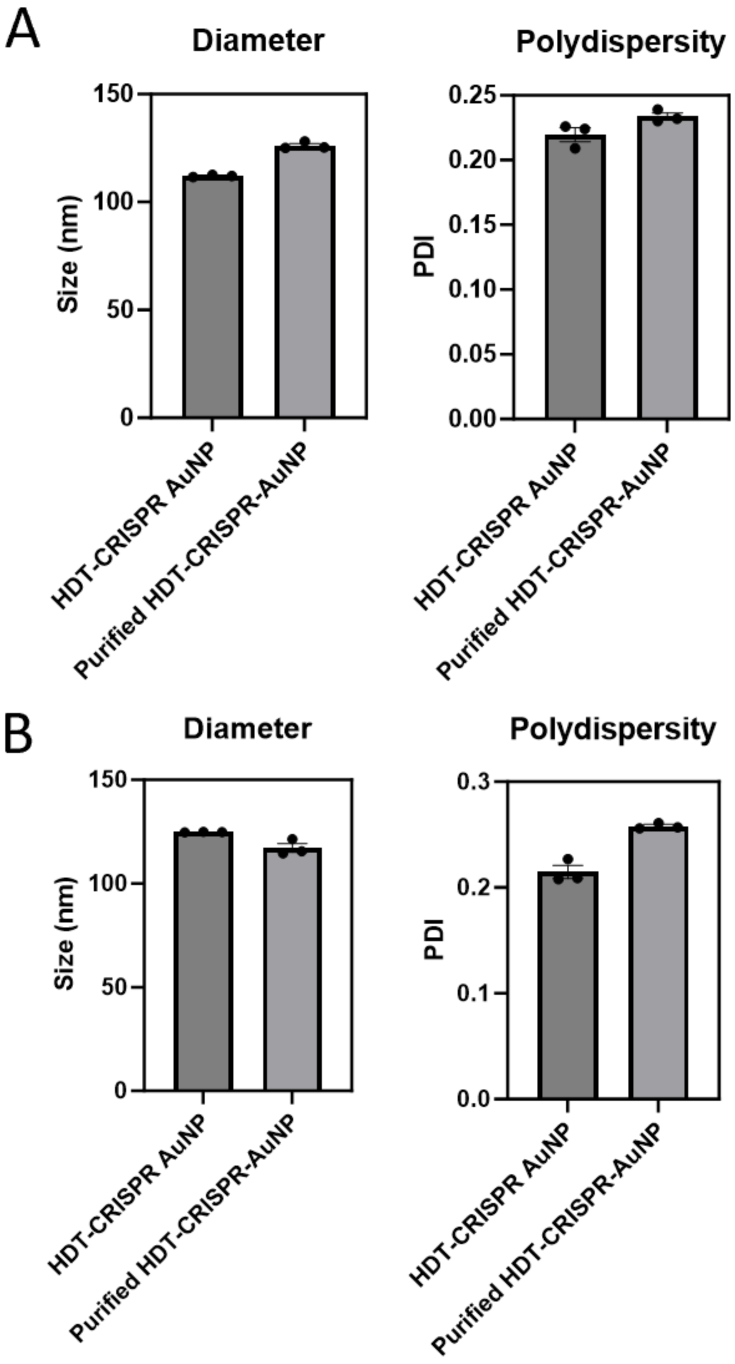

Figure S3 : Integration PCR negative gels and Sanger sequencing analysis of positive bands

A. Integration PCR Gel Images

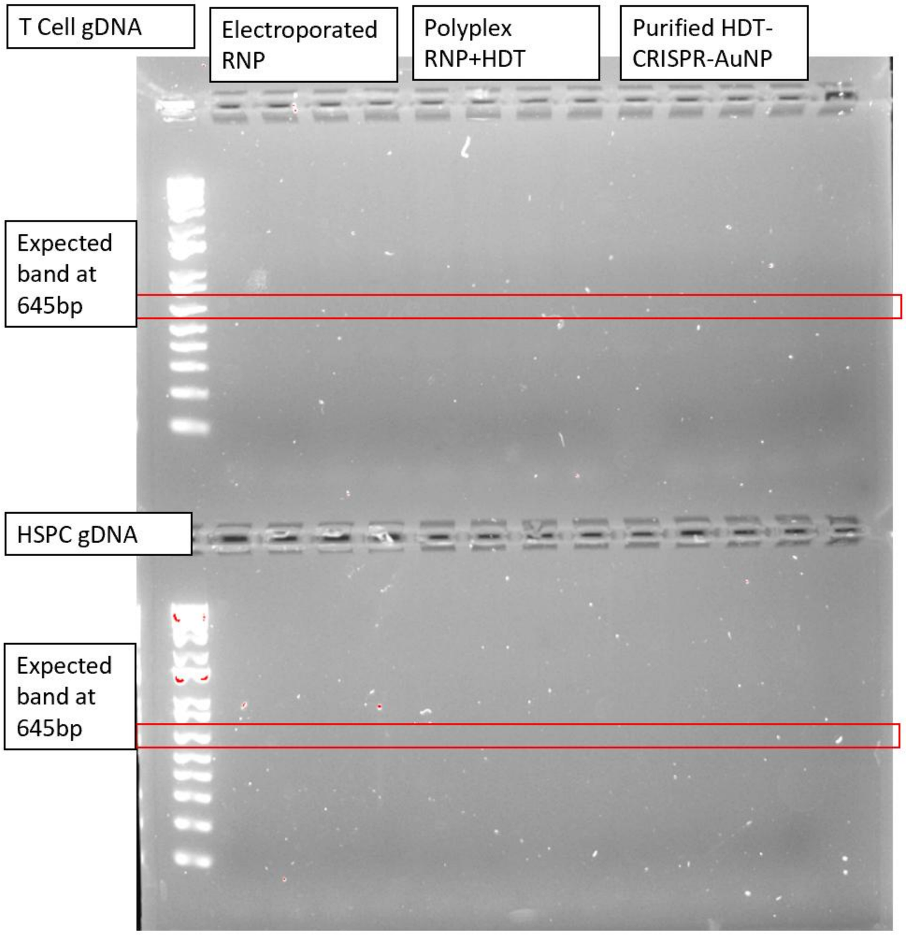

B. Sanger Sequencing Trace Alignments

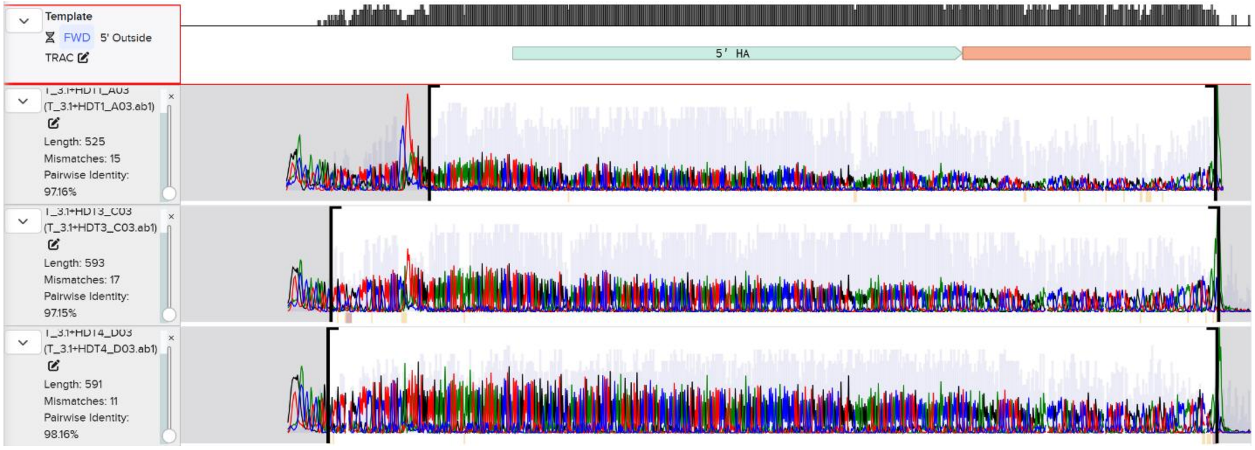

Sanger traces of bands gel extracted from integration PCR show on average 97.49% sequence identity to target sequence of HDT integrated into genome.
